# Autosomal Recessive Nonsyndromic Hearing Loss: A Case Report with a Mutation in *TRIOBP* Gene

**Published:** 2015

**Authors:** Majid Fardaei, Shaghayegh Sarrafzadeh, Soudeh Ghafouri-Fard, Mohammad Miryounesi

**Affiliations:** 1 *Department of Medical Genetics, Shiraz University of Medical Sciences, Shiraz, Iran.*; 2 *Department of Medical Genetics, Shahid Beheshti University of Medical sciences, Tehran, Iran.*; 3 *Genomic Research Center, School of Medicine, Shahid Beheshti University of Medical Sciences, Tehran, Iran.*

**Keywords:** Hearing loss, *TRIOBP*, mutation

## Abstract

Hearing loss (HL) is the most common sensory defect. Various genetic as well as environmental factors have been shown to contribute in it. More than 100 loci have been recognized to cause autosomal recessive nonsyndromic hearing loss (ARNSHL). Here, we report a 6-year old female patient with bilateral pre-lingual HL in whom a mutation has been identified in *TRIOBP* gene (c.6362C>T, S2121L). *In silico* analysis has shown that this variant is possibly pathogenic. Although several mutations have been detected in this gene in various populations, this is the first report identifying *TRIOBP* mutation in Iranian population. Consequently, the results of the present study may be of importance in genetic counseling.

Hearing loss (HL) is the most common sensory defect worldwide with both genetics as well as environmental factors contributing in it. More than half of cases with pre- lingual HL are thought to be caused by genetic changes. In addition, more than 70% of genetic HL cases are nonsyndromic ones with most of them being inherited in an autosomal recessive manner (1). The genetic factors are very diverse and more than 100 loci have been recognized to cause autosomal recessive non-syndromic hearing loss (ARNSHL) (2). In Iran, HL is the second most common genetic disorder after intellectual disability (3). The high incidence of this disorder in Iran is attributed to the high percentage of consanguineous marriages in this region (1). The diverse spectrum of mutations in this genetic disorder makes the genetic counseling very problematic and challenging.

**Case report**

Here, we present a 6-year old female patient with bilateral pre-lingual HL, the first child of consanguineous parents from the southern part of Iran. No other abnormality has been observed in her clinical examination or past medical history. Her parents were clinically normal. To identify the underlying genetic defect, genomic DNA was extracted from blood samples of the patient and her parents after obtaining informed consent using the standard salting out method. Sequence analysis was performed using a custom designed NimbleGen chip capturing of 127 HL related genes including *GJB2*, *GJB6*, *SLC26A4*, *MT-RNR1*, *MT-TS1*, and so on, followed by the next generation sequencing (BGI-Clinical Laboratories, Shenzhen, China). In general, the test platform is claimed to examine >95% of the target genes with sensitivity of more than 99% and can simultaneously detect point mutations, micro-insertions, deletions and duplications.

A homozygous missense mutation, c.6362C>T (S2121L) in *TRIOBP* gene was detected. The results were validated by Sanger sequencing and targeted sequencing on the parents showed the expected segregation pattern (Figure 1). Afterwards, the frequency of the identified variant was checked in the international mutation and polymorphism databases. In addition, *in silico* functional analysis of the sequencing results were performed by bioinformatics tools such as SIFT (Sorting Intolerant From Tolerant), Polyphen -2 (Polymor-phism Phenotyping v2) and Combined Annotation Dependent Depletion (CADD).

## Discussion

The S2121L variant in *TRIOBP* has not been reported in individuals with hearing loss, but has been found in 0.05% (4/8458) of European American people at heterozygous state according to 

**Table 1:** Reported mutations in *TRIOBP* gene

**Ethnicity**	**Pathogenic variant**	**Genomic location**	**Protein effect**	**Reference**
Indian	c.889C>T	Exon 6	p.Gln297X	(4)
Palestinian	c.1039C>T	Exon 6	p.Arg347X	(5)
Palestinian	c.1741C>T	Exon 6	p.Gln581X	(5)
Turkish	c.2355_2356delAG	Exon 6	p.Arg785SerfsTer50	(6)
Pakistani	c.2362C>T	Exon 6	p.Arg788X	(4)
Chinese	c.2581C>T	Exon 6	p.Arg861X	(7)
Chinese	c.2758C>T	Exon 6	p.Arg920X	(7)
Palestinian	c.3055G>A	Exon 6	p.Gly1019Arg	(5)
Pakistani	c.3202C>T	Exon 6	p.Arg1068X	(4)
Indian	c.3202_3203delCG	Exon 6	p.Asp1069fsX1082	(4)
Indian	c.3225_3226insC	Exon 6	p.Arg1078fsX1083	(4)
Indian	c.3349C>T	Exon 6	p.Arg1117X	(4)
Chinese	c.3451A>G	Exon 6	p.Met1151Val	(7)
Chinese	c.4187C>G	Exon 8	p. Arg1396Pro	(7)
Iranian	c.6362C>T	Exon 18	p.Ser2121Leu	The present study

**Fig. 1:**
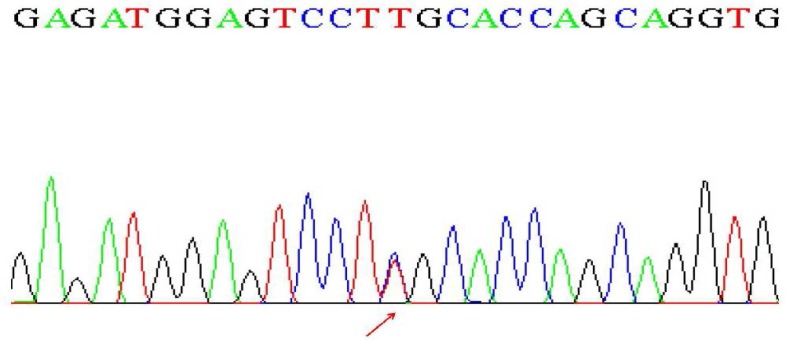
Sequence analysis of the corresponding region in the parents showing heterozygous state.

ClinVar data (http:// www.ncbi.nlm.nih.gov/ clinvar/ varia-tion/ 165609/#suppobsdesc1). The frequency of this variant in normal population as revealed by 1000 genome project data is 0.001. It is predicted to be deleterious by SIFT and possibly damaging by Polyphen-2. In addition, we have used the recently introduced Combined Annotation Dependent Depletion (CADD) (8) method which presents a standardized, genome-wide, variant scoring metric (C-score) integrating SIFT and PolyPhen to predict the pathogenicity of this variant. This tool indicated that this variant would be deleterious with a scaled C-score of 34.

Previously, at least 14 mutations were reported in *TRIOBP* gene in different populations (4, 5). As presented in table 1, nearly all of the previously reported mutations of *TRIOBP* causing HL are located in exon 6. Although it has been demonstrated that *TRIOBP* has different isoforms which are produced through the use of 2 alternate promoters, all previously reported *TRIOBP* mutations only affect the *TRIOBP4* and *TRIOBP5* isoforms (9). *TRIOBP5* is the longest transcript that uses a distal promoter upstream of exon 1 and ends in exon 24. *TRIOBP4* encodes a shorter protein product and terminates after exon 6. TRIOBP5 has been shown to be localized primarily into the stereocilia rootlets, and TRIOBP4 is expressed along the rootlets and the entire stereocilia length (9). The detected mutation in the present study is located in exon 18 which is present in TRIOBP5 but not TRIOBP4 isoform. Although the previously reported mutations were all located in the shared region between TRIOBP5 and TRIOBP4 isoforms, the important function of cells expressing *TRIOBP5* in inner ear implies that other regions of *TRIOBP* may be the targets of mutations in HL. Though several comprehensive studies have been done to explore the range of mutations in ARNSHL in Iranian population (1, 3), to our knowledge this is the first report of *TRIOBP* mutation discovery in this population. Consequently, the results of the present study may be of importance in genetic counseling.

## Conflict of interests

The authors declared no conflict of interests.
